# Performance of Prognostic Scoring Systems in Trauma Patients in the Intensive Care Unit of a Trauma Center

**DOI:** 10.3390/ijerph17197226

**Published:** 2020-10-02

**Authors:** Shao-Chun Wu, Sheng-En Chou, Hang-Tsung Liu, Ting-Min Hsieh, Wei-Ti Su, Peng-Chen Chien, Ching-Hua Hsieh

**Affiliations:** 1 Department of Anesthesiology Kaohsiung Chang Gung Memorial Hospital, Chang Gung University College of Medicine, Kaohsiung 803, Taiwan; shaochunwu@gmail.com; 2 Department of Trauma Surgery, Kaohsiung Chang Gung Memorial Hospital, Chang Gung University College of Medicine, Kaohsiung 803, Taiwan; athenechou@gmail.com (S.-E.C.); htl1688@yahoo.com.tw (H.-T.L.); hs168hs168@gmail.com (T.-M.H.); s101132@adm.cgmh.org.tw (W.-T.S.); 3 Department of Plastic Surgery, Kaohsiung Chang Gung Memorial Hospital, Chang Gung University College of Medicine, Kaohsiung 803, Taiwan; VENU_CHIEN@hotmail.com

**Keywords:** trauma, trauma and injury severity score, intensive care unit, mortality, prognostic scoring systems

## Abstract

Background: Prediction of mortality outcomes in trauma patients in the intensive care unit (ICU) is important for patient care and quality improvement. We aimed to measure the performance of 11 prognostic scoring systems for predicting mortality outcomes in trauma patients in the ICU. Methods: Prospectively registered data in the Trauma Registry System from 1 January 2016 to 31 December 2018 were used to extract scores from prognostic scoring systems for 1554 trauma patients in the ICU. The following systems were used: the Trauma and Injury Severity Score (TRISS); the Acute Physiology and Chronic Health Evaluation (APACHE II); the Simplified Acute Physiology Score (SAPS II); mortality prediction models (MPM II) at admission, 24, 48, and 72 h; the Multiple Organ Dysfunction Score (MODS); the Sequential Organ Failure Assessment (SOFA); the Logistic Organ Dysfunction Score (LODS); and the Three Days Recalibrated ICU Outcome Score (TRIOS). Predictive performance was determined according to the area under the receiver operator characteristic curve (AUC). Results: MPM II at 24 h had the highest AUC (0.9213), followed by MPM II at 48 h (AUC: 0.9105). MPM II at 24, 48, and 72 h (0.8956) had a significantly higher AUC than the TRISS (AUC: 0.8814), APACHE II (AUC: 0.8923), SAPS II (AUC: 0.9044), MPM II at admission (AUC: 0.9063), MODS (AUC: 0.8179), SOFA (AUC: 0.7073), LODS (AUC: 0.9013), and TRIOS (AUC: 0.8701). There was no significant difference in the predictive performance of MPM II at 24 and 48 h (*p* = 0.37) or at 72 h (*p* = 0.10). Conclusions: We compared 11 prognostic scoring systems and demonstrated that MPM II at 24 h had the best predictive performance for 1554 trauma patients in the ICU.

## 1. Introduction

Predicting mortality in trauma patients in the intensive care unit (ICU) is important for planning better treatment and improving the overall quality of patient care. The Trauma and Injury Severity Score (TRISS) is the most commonly used prediction algorithm to predict mortality outcomes in trauma patients [1,2,3]. The TRISS determines the probability of survival mainly using four variables—age; the Injury Severity Score (ISS), an anatomical variable; the Revised Trauma Score (RTS), a physiological variable value related to the patient’s initial Glasgow Coma Scale (GCS) score; systolic blood pressure (SBP); and respiratory rate (RR) [4]—and the injury mechanism, such as blunt or penetrating injuries. However, there is still room for improvement in prediction accuracy based on anatomical and physiological injury scores alone [5]. Therefore, adding clinical data such as previous health status, the main diagnosis of acute illness, physiological change, and laboratory data has been recommended to improve the accuracy of TRISS [6,7].

Many prognostic scoring systems have been developed for critically ill patients in the ICU. Scores for the following prognostic scoring systems are calculated using data collected on the first day in the ICU: the Acute Physiology and Chronic Health Evaluation (APACHE II) [8], the Simplified Acute Physiology Score (SAPS II) [9], and the Mortality Prediction Model (MPM II) at admission [10]. Scores from the following prognostic scoring systems are calculated using data collected from the first day in the ICU until the patient’s departure from the ICU, or for the first three days: MPM II at 24, 48, and 72 h [10], the Multiple Organ Dysfunction Score (MODS) [11], the Sequential Organ Failure Assessment (SOFA) [12], the Logistic Organ Dysfunction Score (LODS) [13], and the Three Days Recalibrated ICU Outcome Score (TRIOS) [14]. Although the performance of these systems has been extensively validated in the literature for patients in the ICU, these systems are commonly used for general patients with critical illness. Different scoring systems vary in their predictions according to the diagnoses [15]. For example, the Revised Injury Severity Classification II (RISC II) score is mainly based on severely injured patients treated in the ICU [16], the Emergency Surgery Score (ESS) is recommended for triaging perioperative patients [17], and the Physiological Parameters for Prognosis in Abdominal Sepsis (PIPAS) is for patients with acute peritonitis [18]. The scoring systems currently in use may have varied results for trauma patients [19,20]. As the best predictive score should be validated in the focused population and geographic region where the scoring system is to be employed [6], this study was designed to compare the performance of the aforementioned 11 prognostic scoring systems for predicting mortality outcomes in trauma patients in the ICU. This study was performed based on prospectively registered data in the Trauma Registry System of Kaohsiung Chang Gung Memorial Hospital over a three-year period.

## 2. Materials and Methods

### 2.1. Study Population and Data Collection

This study was approved (approval numbers: 201901360B0 and 201900298B0) by the Institutional Review Board (IRB) of Kaohsiung Chang Gung Memorial Hospital, a 2686-bed level I trauma center in Southern Taiwan [21,22,23]. The informed consent requirement was waived in accordance with IRB regulations. Detailed information on trauma patients who were admitted to the ICU between 1 January, 2016 and 31 December, 2018 that were prospectively registered in the hospital’s Trauma Registry System was retrospectively retrieved for analysis. Information was collected about age, sex, body mass index (BMI), pre-existing comorbidities (diabetes mellitus (DM), hypertension (HTN), coronary artery disease (CAD), congestive heart failure (CHF), cerebral vascular accident (CVA), and end-stage renal disease (ESRD)), the Abbreviated Injury Scale (AIS) scores in different regions of the body (head, face, thorax, abdomen, extremities, and external regions), the ISS, and the TRISS. Vital signs (temperature, SBP, diastolic blood pressure, mean arterial pressure, heart rate (HR), and RR), and GCS scores were recorded at triage on arrival at the emergency department. Laboratory data at the emergency room, including sodium (Na) levels, potassium (K) levels, blood urine nitrogen (BUN) levels, creatinine (Cr) levels, bilirubin levels, white blood cell (WBC) counts, hematocrit (Hct) levels, platelet counts, and blood gas levels (oxygenation, arterial pH, and bicarbonate (HCO_3_)) were recorded. In-hospital mortality was recorded as the primary outcome for prediction. TRISS was calculated based on a logarithmic regression equation: survival probability = 1/(1 + e − b), where b (penetrating injury) = −2.5355 + 0.9934 × RTS − 0.0651 × ISS − 1.1360 × Age (index) and b (blunt injury) = −0.4499 + 0.8085 × RTS − 0.0835 × ISS − 1.7430 × Age (index). In the formula, the Age (index) was awarded 1 for patients above the age of 55, and 0 for patients at or below the age of 55 [4]. The APACHE II, SAPS II, and MPM II scores were calculated according to the variables recorded at admission. The scores for MPM II at 24, 48, and 72 h and MODS, SOFA, LODS, and TRIOS were calculated according to the original proposed algorithms [24].

### 2.2. Statistical Analyses

All statistical analyses were performed using SPSS for Windows version 23.0 (IBM Inc., Chicago, IL, USA) or R 3.3.3. The Chi-square test was used to determine the significance of the association between categorical variables. The Kolmogorov–Smirnov test was used to analyze the normalization of the distributed data for continuous variables. The abnormally distributed data were analyzed using the Mann–Whitney U test. The results are presented as median ± interquartile range (IQR). Predictive performance was determined according to the area under the receiver operating characteristic curve (AUC) using the roc and roc.test function in the pROC package in R [25]. Because the TRISS measures the probability of survival, 1 − TRISS was used to present the probability of mortality for a patient while plotting the receiver operating characteristic curves. A *p*-value of <0.05 was considered statistically significant. Calibration curves were plotted to determine the degree of agreement between the observed outcomes and predicted probabilities of each model by calculating the rank correlation coefficient of Somers’ Dxy, the c-index, *R*^2^, and the Brier score. Somers’ Dxy determines the predictive discrimination with measured probability of concordance minus the probability of discordance between predicted and observed outcomes. The c-index describes how well the model is able to discriminate between mortal and non-mortal patients; a c-index score >0.9 indicates outstanding discrimination. *R*^2^ quantifies the goodness-of-fit of a model [26], with *R*^2^ = 1 indicating that the regression line fits the data perfectly. The Brier score is defined as the mean squared difference between the actual outcome and the predicted probability and falls in the range between 0 and 1 [27]. A lower Brier score indicates a better calibrated prediction.

## 3. Results

### 3.1. Patient Demographics

**Figure 1:**
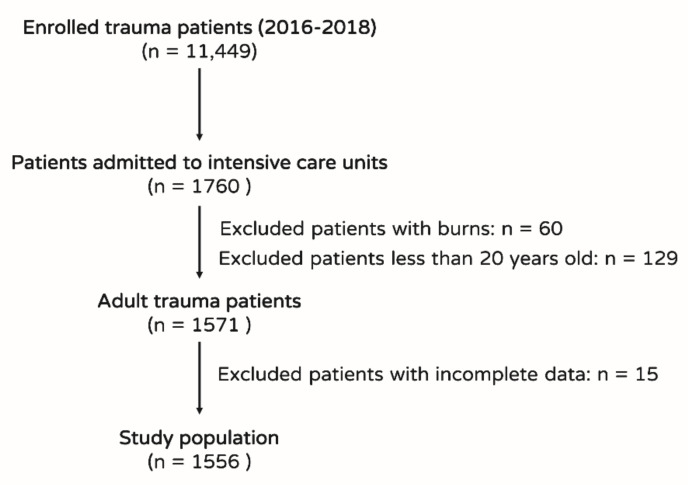
Flow chart showing the selection of trauma patients in the intensive care unit (ICU) for the study population.

As shown in the flow chart in Figure 1, of the 11,449 enrolled trauma patients, 1760 patients were admitted to the ICU. After excluding 60 patients with burns, 129 patients younger than 20 years, and 15 patients with incomplete data, 1554 patients were left in the study population. Among the 1554 patients enrolled, 178 patients died and 1376 patients survived. Patients who died had higher incidence of pre-existing HTN, CAD, and ESRD and higher AIS scores in the head and thorax regions than those who survived (Table 1). There were no significant differences in sex; pre-existing DM, CHF, or CVA; or AIS scores in the face, abdomen, extremities, or external regions between patients who died and those who survived. Patients who died were significantly older; had higher heart rates, and lower body temperatures, blood pressures, and respiratory rates; and worse GCS scores, ISS, and renal function (BUN level, Cr level, and urine output) than those who survived. Patients who died also had significantly lower levels of Hct, platelets, arterial pH, and HCO_3_ than those who survived (Table 2). There were no significant differences in BMI, Na levels, K levels, bilirubin levels, WBC counts, and oxygenation levels between patients who died and those who survived (Table 2). Patients who died had significantly shorter stays in the hospital than those who survived (median IQR: 5 days [1,14] vs. 13 days [7,22], *p* < 0.001). Patients who died had significantly lower TRISS and higher APACHE II; SAPS II; MPM II at admission and 24, 48, and 72 h; and MODS, SOFA, LODS, and TRIOS scores than those who survived (Table 3).

### 3.2. Performance of the Prognostic Scoring Systems

**Figure 2:**
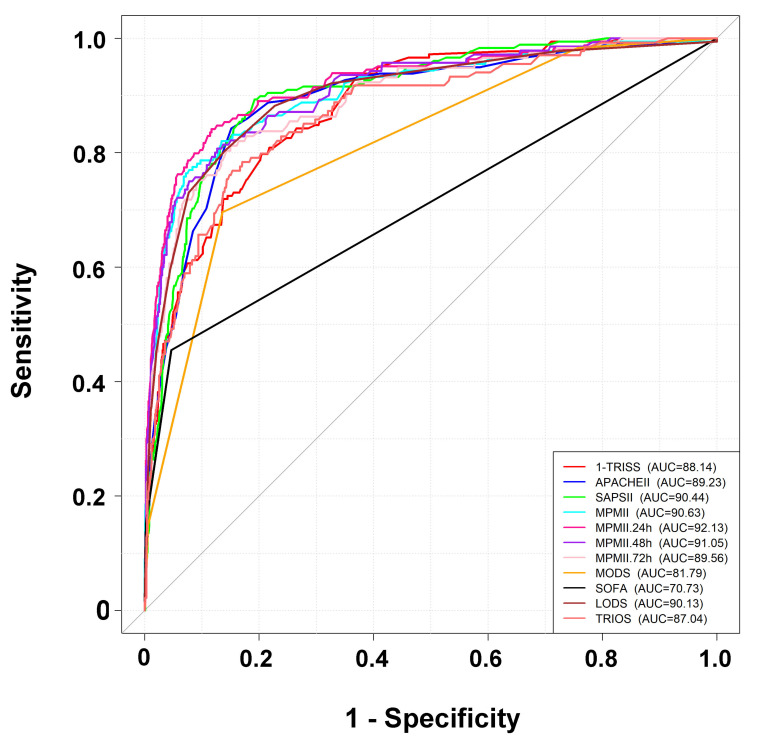
The receiver operating characteristic curves and the area under the curve (AUC) of the prognostic scoring systems for trauma patients with critical illness.

A comparison of AUCs among the 11 prognostic scoring systems (Figure 2) demonstrated that the MPM II at 24 h had the highest AUC (0.9213), followed by the MPM II at 48 h (AUC: 0.9105) (Table 4). The MPM II had a significantly higher AUC at 24, 48, and 72 h than at admission (AUC: 0.9063). There was no significant difference in the predictive performance of the MPM II at 24 and 48 h (*p* = 0.37) or at 72 h (*p* = 0.10). The TRISS, APACHE II, SAPS II, LODS, and TRIOS systems had an AUC of 0.8814, 0.8923, 0.9044, 0.9013, and 0.8701, respectively. The MODS (AUC: 0.8179) and SOFA (AUC: 0.7073) systems had a significantly lower predictive performance than the other prognostic scoring systems.

**Figure 3:**
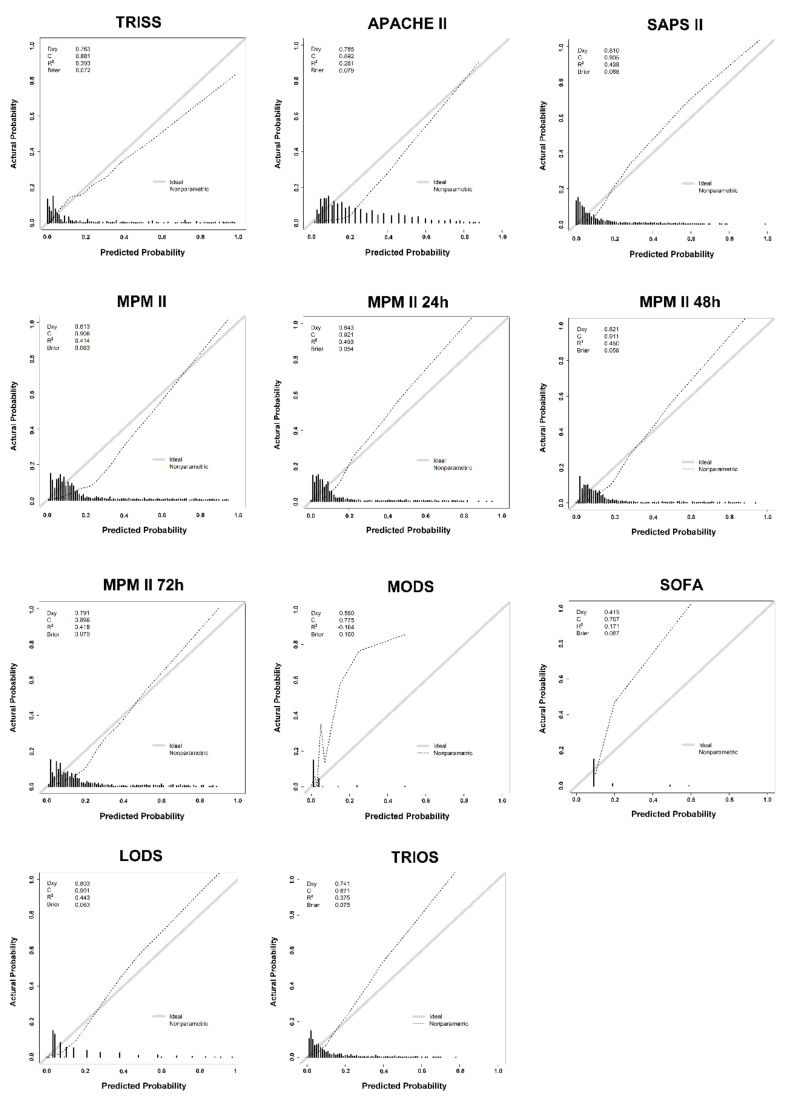
The calibration curves of the prognostic scoring systems for trauma patients with critical illness.

The calibration curves of these eleven predictions are demonstrated in Figure 3. The MPM II at 24 h generated a nonparametric line close to the ideal diagonal line with the highest Somers’ Dxy (0.843), c-index (0.921), and *R*^2^ (0.493) and the lowest Brier score (0.054), followed by the MPM II at 48 h (Somers’ Dxy: 0.821, c-index: 0.911, *R*^2^: 0.450, and Brier score: 0.059). By contrast, MODS, SOFA, and TRIOS exhibited marked deviation from the ideal diagonal line located between the predicted probability and the actual outcome.

## 4. Discussion

In this study, we compared the performance of 11 prognostic scoring systems for predicting mortality outcomes in trauma patients in the ICU and revealed that the MPM II has the best predictive performance. The MPM II at 24, 48, and 72 h had a significantly higher AUC than all the other scoring systems. In addition, there was no significant difference in the predictive performance of the MPM II at 24, 48, or 72 h.

The MPM II uses data on heath condition (medical or unscheduled surgical admission), pre-existing illness (such as metastatic neoplasm and cirrhosis), acute diagnosis (such as infection, coma, and intracranial mass effect), physiological variables (such as Cr levels, urine output, and partial pressure of oxygen), laboratory data (prothrombin time), and other variables (such as mechanical ventilation and use of vasoactive drugs) [10]. The MPM II at 48 and 72 h uses the same variables as at 24 h and is based on the most deranged values of the preceding 24 h to determine the outcome with different weights from logistic regression [28,29]. Because the physiological variables of patients are dynamic and may be influenced by ongoing management and resuscitation, estimating the outcome based only on physiological variables would lead to bias [19]. Differences in the variables used in different systems (e.g., acute diagnosis is a variable in APACHE II, but not in SAPS II) would contribute to discrepancies in the performance of these systems [30]. Hence, in our study, better performance of the MPM II than the MODS, LODS, and TRIOS is in line with expectations because the MODS and LODS use only physiological and laboratory data and the TRIOS uses only daily SAPS II and LODS data. In the SOFA system, physiological or laboratory variables from five organ systems are classified by integer from 0 to 4, but the real number for computing also markedly reduces its performance in the prediction of mortality. Furthermore, not using laboratory data in the TRISS reduces its performance.

When choosing a scoring system for specific populations in the ICU, the performance, feasibility (e.g., time to calculate score, abstraction burden, copyright), and interobserver variability should be considered [6]. For a prognostic model to be effective for critical care patients, an acceptable time for data collection is needed. The MPM II uses data exclusively obtained at the time of ICU admission as their proponents have focused on simplicity and feasibility for routine use. The MPM II has the lowest abstraction burden and is less prone to interobserver variability because it uses less physiological and laboratory data [31]. In contrast, the use of APACHE II is deterred by the possible associated comorbidities; furthermore, the selection of only one principal diagnostic category from many specific acute diagnoses may be very difficult [24]. The abstraction burden of the APACHE II is substantially greater than that of the MPM II [32].

The study had a few limitations. First, patients declared dead on arrival at the emergency room were not recorded in the registered database. In addition, only in-hospital mortality, not long-term mortality, was evaluated. This may have led to a selection bias. Second, the lack of data on the mechanism of trauma or severity of injury, which are generally used in the assessment of trauma patients, may have limited the accuracy of the prediction systems for trauma patients. Third, because the effects of any one particular treatment intervention could not be assessed, especially the use of vasopressive support on admission and surgical interventions, we assumed that the treatment outcomes were uniform across all the patients studied. Finally, only single-center data from southern Taiwan was used in the analysis; hence, the study results may not be applicable for other populations.

## 5. Conclusions

This study revealed that the MPM II at 24 h had the best predictive performance for predicting mortality outcomes in 1554 trauma patients in the ICU after comparing the performance of the 11 most popular prognostic scoring systems. Considering that each scoring system contains different variables possibly related to the patient’s outcome, further study to update some of these prognostic scores is encouraged to make them more usable for trauma populations.

## Figures and Tables

**Table 1:** Categorical variables of characteristics of critically ill trauma patients who died or survived.

Variables		Total	Mortality		*p*-Value
		(n = 1554)	No (n = 1376)	Yes (n = 178)	
Sex	Female	529 (34.0%)	478 (34.7%)	51 (28.7%)	0.111
	Male	1025 (66.0%)	898 (65.3%)	127 (71.4%)	
Diabetes mellitus (DM)	No	1257 (80.9%)	1121 (81.5%)	136 (76.4%)	0.106
	Yes	297 (19.1%)	255 (18.5%)	42 (23.6%)	
Hypertension (HTN)	No	1023 (65.8%)	924 (67.2%)	99 (55.6%)	0.003
	Yes	531 (34.2%)	452 (32.9%)	79 (44.4%)	
Coronary artery disease (CAD)	No	1423 (91.6%)	1274 (92.6%)	149 (83.7%)	<0.001
	Yes	131 (8.4%)	102 (7.4%)	29 (16.3%)	
Congestive heart failure (CHF)	No	1548 (99.6%)	1372 (99.7%)	176 (98.9%)	0.144
	Yes	6 (0.4%)	4 (0.3%)	2 (1.1%)	
Cerebral vascular accident (CVA)	No	1464 (94.2%)	1299 (94.4%)	165 (92.7%)	0.392
	Yes	90 (5.8%)	77 (5.6%)	13 (7.3%)	
End-stage renal disease (ESRD)	No	1507 (97.0%)	1343 (97.6%)	164 (92.1%)	<0.001
	Yes	47 (3.0%)	33 (2.4%)	14 (7.9%)	
Abbreviated Injury Scale (AIS, Head)	0	403 (25.9%)	384 (27.9%)	19 (10.7%)	<0.001
	1	18 (1.2%)	18 (1.3%)	0 (0.0%)	
	2	110 (7.1%)	108 (7.9%)	2 (1.1%)	
	3	524 (33.7%)	511 (37.1%)	13 (7.3%)	
	4	270 (17.4%)	236 (17.2%)	34 (19.1%)	
	5	223 (14.4%)	118 (8.6%)	105 (59.0%)	
	6	6 (0.4%)	1 (0.1%)	5 (2.8%)	
AIS (Face)	0	1265 (81.4%)	1118 (81.3%)	147 (82.6%)	0.939
	1	56 (3.6%)	49 (3.6%)	7 (3.9%)	
	2	224 (14.4%)	201 (14.6%)	23 (12.9%)	
	3	9 (0.6%)	8 (0.6%)	1 (0.6%)	
AIS (Thorax)	0	1066 (68.6%)	958 (69.6%)	108 (60.7%)	<0.001
	1	42 (2.7%)	39 (2.8%)	3 (1.7%)	
	2	116 (7.5%)	101 (7.3%)	15 (8.4%)	
	3	288 (18.5%)	249 (18.1%)	39 (21.9%)	
	4	23 (1.5%)	14 (1.0%)	9 (5.1%)	
	5	19 (1.2%)	15 (1.1%)	4 (2.3%)	
AIS (Abdomen)	0	1271 (81.8%)	1120 (81.4%)	151 (84.8%)	0.055
	2	108 (7.0%)	99 (7.2%)	9 (5.1%)	
	3	86 (5.5%)	83 (6.0%)	3 (1.7%)	
	4	61 (3.9%)	51 (3.7%)	10 (5.6%)	
	5	28 (1.8%)	23 (1.7%)	5 (2.8%)	
AIS (Extremities)	0	1004 (64.6%)	890 (64.7%)	114 (64.0%)	0.833
	1	9 (0.6%)	8 (0.6%)	1 (0.6%)	
	2	364 (23.4%)	323 (23.5%)	41 (23.0%)	
	3	157 (10.1%)	139 (10.1%)	18 (10.1%)	
	4	20 (1.3%)	16 (1.2%)	4 (2.3%)	
AIS (External)	0	1404 (90.4%)	1235 (89.8%)	169 (94.9%)	0.168
	1	87 (5.6%)	84 (6.1%)	3 (1.7%)	
	2	32 (2.1%)	29 (2.1%)	3 (1.7%)	
	3	11 (0.7%)	11 (0.8%)	0 (0.0%)	
	4	6 (0.4%)	5 (0.4%)	1 (0.6%)	
	5	14 (0.9%)	12 (0.9%)	2 (1.1%)	

**Table 2:** Continuous variables of characteristics of critically ill trauma patients who died or survived.

Variables	Total	Mortality		*p*-Value
	(n = 1554)	No (n = 1376)	Yes (n = 178)	
Age (years)	58 (41,71)	57 (40,71)	65 (49,76)	<0.001
Body mass index (BMI)	23.6 (21.0, 26.6)	23.5 (20.9, 26.6)	23.7 (21.1, 26.6)	0.575
Temperature (°C)	37 (36,37)	36.8 (36.2, 37.4)	36.0 (35.0, 37.1)	<0.001
Systolic blood pressure (SBP, mmHg)	140 (123,156)	141 (125,156)	132 (105,155)	<0.001
Diastolic blood pressure (DBP, mmHg)	77 (66,88)	78 (67,88)	71 (56,83)	<0.001
Mean arterial pressure (MAP, mmHg)	98 (87,109)	99 (88,109)	93 (74,105)	<0.001
Heart rate (times/min)	89 (77,102)	88 (76,101)	99 (84,117)	<0.001
Respiratory rate (times/min)	18 (15,21)	18 (15,21)	16 (13,21)	0.009
Glasgow Coma Scale (GCS)	13 (9,15)	14 (10,15)	4 (3,7)	<0.001
Injury Severity Score (ISS)	16 (9,24)	16 (9,20)	25 (25,34)	<0.001
Sodium (Na, mEq/L)	139 (137,140)	139 (137,140)	138 (136,141)	0.585
Potassium (K, mEq/L)	4 (3,4)	3.7 (3.3, 4.0)	3.7 (3.2, 4.1)	0.667
Blood urine nitrogen (BUN, mg/dL)	14 (11,19)	14 (11,19)	16 (12,24)	<0.001
Creatinine (Cr, mg/dL)	0.94 (0.75, 1.19)	0.92 (0.74, 1.14)	1.16 (0.92, 1.64)	<0.001
Bilirubin (mg/dL)	0.8 (0.6, 1.1)	0.8 (0.6, 1.1)	0.8 (0.5, 1.1)	0.937
White Blood Cell (WBC, 10^3^/uL)	12.6 (9.0, 17.4)	12.6 (8.9, 17.5)	12.4 (9.7, 16.6)	0.948
Hematocrit (Hct, %)	38.2 (33.8, 42.0)	38.6 (34.4, 42.0)	34.9 (30.2, 40.5)	<0.001
Platelets (10^3^/uL)	199 (160,242)	203 (163,247)	176 (127,216)	<0.001
Oxygenation (mmHg)	121.8 (84.5, 176.1)	120.9 (85.7, 170.9)	130.3 (80.7, 203.2)	0.234
Arterial pH	7.4 (7.3, 7.4)	7.4 (7.4, 7.4)	7.4 (7.3, 7.4)	<0.001
HCO_3_ (meq/L)	21.8 (19.4, 23.6)	22.1 (19.7, 23.8)	20.4 (17.0, 22.4)	<0.001
Urine output (L/day)	2.0 (1.5, 2.7)	2.1 (1.5, 2.7)	1.8 (1.0, 2.8)	0.001
Length of stay in hospital (days)	12 (7,22)	13 (7,22)	5 (1,14)	<0.001

**Table 3:** Comparison of the prognostic scoring systems in critically ill trauma patients who died or survived.

Variables	Total	Mortality		*p*-Value
	(n = 1554)	No (n = 1376)	Yes (n = 178)	
Trauma and Injury Severity Score (TRISS)	0.96 (0.89, 0.98)	0.97 (0.91, 0.98)	0.61 (0.27, 0.89)	<0.001
Acute Physiology and Chronic Health Evaluation (APACHE II)	12 (8,18)	11 (7,16)	24 (20,29)	<0.001
Simplified Acute Physiology Score (SAPS II)	24 (17,35)	23 (15,31)	52 (41,60)	<0.001
Mortality prediction model (MPM II)	10.8 (6.6, 19.2)	10.0 (6.2, 15.3)	59.3 (35.7, 76.5)	<0.001
MPM II 24 h	7.0 (4.0, 11.7)	6.4 (3.7, 10.0)	47.3 (21.9, 65.1)	<0.001
MPM II 48 h	8.8 (5.1, 14.9)	8.0 (4.6, 12.9)	49.3 (22.7, 67.2)	<0.001
MPM II 72 h	11.1 (6.0, 19.2)	9.6 (5.7, 15.9)	51.3 (25.8, 69.1)	<0.001
Multiple Organ Dysfunction Score (MODS)	2 (1,4)	2 (1,3)	6 (4,8)	<0.001
Sequential Organ Failure Assessment (SOFA)	2 (1,4)	2 (1,4)	6 (4,9)	<0.001
Logistic Organ Dysfunction Score (LODS)	2 (1,4)	1 (0,3)	7 (5,9)	<0.001
Three Days Recalibrated ICU Outcome Score (TRIOS)	6.5 (3.1, 14.1)	5.9 (2.9, 10.7)	27.3 (16.5, 46.2)	<0.001

**Table 4:** Comparison of the predictive performance of the evaluated prognostic scoring systems for trauma patients with critical illness.

Performance	The Area under the Curve (AUC) of Receiver Operating Characteristic (ROC) Curves										
	88.14%	89.23%	90.44%	90.63%	92.13%	91.05%	89.56%	81.79%	70.73%	90.13%	87.04%
Variables	TRISS	APACHE II	SAPS II	MPM II	MPM II 24 h	MPM II 48 h	MPM II 72 h	MODS	SOFA	LODS	TRIOS
TRISS	-	0.41	0.07	0.04	<0.001	<0.001	<0.001	<0.001	<0.001	0.12	0.13
APACHE II	-	-	0.17	0.09	<0.001	<0.001	<0.001	<0.001	<0.001	0.37	0.13
SAPS II	-	-	-	0.79	0.01	0.02	<0.001	<0.001	<0.001	0.74	0.75
MPM II	-	-	-	-	<0.001	0.01	<0.001	<0.001	<0.001	0.59	0.54
MPM II 24 h	-	-	-	-	-	0.37	0.10	<0.001	<0.001	0.03	0.01
MPM II 48 h	-	-	-	-	-	-	0.01	<0.001	<0.001	0.01	0.01
MPM II 72 h	-	-	-	-	-	-	-	<0.001	<0.001	<0.001	<0.001
MODS	-	-	-	-	-	-	-	-	<0.001	<0.001	<0.001
SOFA	-	-	-	-	-	-	-	-	-	<0.001	<0.001
LODS	-	-	-	-	-	-	-	-	-	-	0.46
